# Copy Number Variation in Inflammatory Breast Cancer

**DOI:** 10.3390/cells12071086

**Published:** 2023-04-04

**Authors:** Aditi Hazra, Andrea O’Hara, Kornelia Polyak, Faina Nakhlis, Beth T. Harrison, Antonio Giordano, Beth Overmoyer, Filipa Lynce

**Affiliations:** 1 Division of Preventive Medicine, Brigham and Women’s Hospital, Harvard Medical School, Boston, MA 02215, USA; 2 Inflammatory Breast Cancer Program, Dana-Farber Cancer Institute, Boston, MA 02115, USA; 3 Bionano Genomics, San Diego, CA 92121, USA; 4 Department of Medical Oncology, Breast Oncology Center, Dana-Farber Cancer Institute, Harvard Medical School, Boston, MA 02115, USA; 5 Department of Surgery, Division of Breast Surgery, Brigham and Women’s Hospital, Harvard Medical School, Boston, MA 02115, USA; 6 Department of Pathology, Brigham and Women’s Hospital, Harvard Medical School, Boston, MA 02115, USA

**Keywords:** inflammatory breast cancer, copy number variation, copy number alterations, multi-omics, subtype, triple-negative, chromosome 7p11, *EGFR*

## Abstract

Identification of a unique genomic biomarker in de novo inflammatory breast cancer (IBC) may provide an insight into the biology of this aggressive disease. The goal of our study was to elucidate biomarkers associated with IBC. We examined breast biopsies collected from Dana–Farber Cancer Institute patients with IBC prior to initiating preoperative systemic treatment (30 samples were examined, of which 14 were eligible). Patients without available biopsies (*n* = 1), with insufficient tumor epithelial cells (*n* = 10), or insufficient DNA yield (*n* = 5) were excluded from the analysis. Molecular subtype and tumor grade were abstracted from a medical records’ review. Ten IBC tumors were estrogen-receptor-positive (ER+) and human epidermal growth factor receptor 2 (HER2)-negative (*n* = 10 out of 14). Sufficient RNA and DNA were simultaneously extracted from 14 biopsy specimens using the Qiagen AllPrep Kit. RNA was amplified using the Sensation kit and profiled using the Affymetrix Human Transcriptome Array 2.0. DNA was profiled for genome-wide copy number variation (CNV) using the Affymetrix OncoScan Array and analyzed using the Nexus Chromosome Analysis Suite. Among the 14 eligible samples, we first confirmed biological concordance and quality control metrics using replicates and gene expression data. Second, we examined CNVs and gene expression change by IBC subtype. We identified significant CNVs in IBC patients after adjusting for multiple comparisons. Next, to assess whether the CNVs were unique to IBC, we compared the IBC CNV data to fresh-frozen non-IBC CNV data from The Cancer Genome Atlas (*n* = 388). On chromosome 7p11.2, we identified significant CN gain located at position 58,019,983-58,025,423 in 8 ER+ IBC samples compared to 338 non-IBC ER+ samples (region length: 5440 bp gain and 69,039 bp, False Discovery Rate (FDR) *p*-value = 3.12 × 10^−10^) and at position 57,950,944–58,025,423 in 3 TN-IBC samples compared to 50 non-IBC TN samples (74,479 base pair, gain, FDR *p*-value = 4.27 × 10^−5^; near the *EGFR* gene). We also observed significant CN loss on chromosome 21, located at position 9,648,315–9,764,385 (*p*-value = 4.27 × 10^−5^). Secondarily, differential gene expression in IBC patients with 7p11.2 CN gain compared to SUM149 were explored after FDR correction for multiple testing (*p*-value = 0.0016), but the results should be interpreted with caution due to the small sample size. Finally, the data presented are hypothesis-generating. Validation of CNVs that contribute to the unique presentation and biological features associated with IBC in larger datasets may lead to the optimization of treatment strategies.

## 1. Introduction

Inflammatory breast cancer (IBC) is the most lethal form of breast cancer. Although IBC is rare, with a 2.5% incidence in the United States [1], 30% of these patients present with distant metastasis [2] at the time of diagnosis. IBC patients account for approximately 8%–10% of breast cancer mortality in the United States [3]. The clinical presentation of IBC includes rapid onset (less than 6 months) of affected breast skin erythema and edema (peau d’orange of the skin), with or without swelling of the entire affected breast [4]. Patients with IBC have a 5-year overall survival rate of only 40.5% compared with 85% in stage III non-IBC [5]. At a molecular level, IBC is characterized by invasiveness and angiogenic ability, fast progression, and a high propensity to disseminate in the dermal lymphatic system and metastasize to distant organs. Mutations [6,7,8], receptor driven gene clusters [9], differential gene expression [10,11], and immune infiltration [12,13,14] have been identified in IBC. Potential copy number gains were identified for 24 potential candidate IBC genes using high-resolution 244 k Agilent Microarrays in 49 IBC patients compared to 124 non-IBC patients suggesting genomic instability in IBC [15,16]. To date, no genomic diagnostic or prognostic biomarkers underlying the clinicopathologic manifestations of IBC have been robustly identified across studies [4,17,18]. Genomic studies using IBC samples collected after treatment may be confounded by the stage and treatment received. In this observational study, we evaluated copy number variation (CNV) in biospecimens from patients with stage III IBC collected prior to the initiation of preoperative systemic treatment compared to fresh-frozen non-IBC biospecimens in The Cancer Genome Atlas (TCGA).

## 2. Materials and Methods

### 2.1. IBC Patient Population

**Figure 1:**
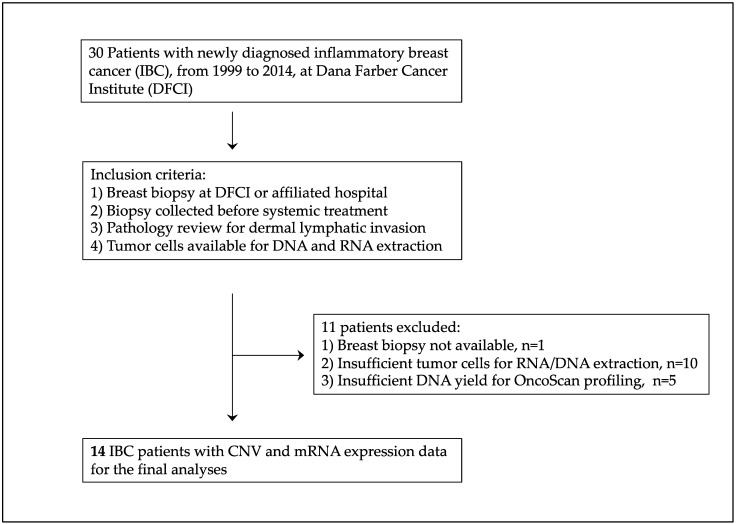
Flow Diagram of the Inflammatory Breast Cancer (IBC) Study Population.

This was an observational analysis of patients who met clinical and pathological criteria (stage T4d) for the diagnosis of IBC. Participating patients were seen at the Inflammatory Breast Cancer Program at the Dana–Farber Cancer Institute (DFCI) between 1999 and 2014 [19]. This study was approved by the Institutional Review Board (IRB) at the DFCI (Protocol 93-085 and DFCI 11-035, Outcomes of IBC). We examined data from 30 IBC patients collected from the electronic medical record (EMR) system and REDCap survey. Formalin-fixed paraffin-embedded (FFPE) core biopsy specimens were obtained from 29 patients with IBC prior to initiating systemic therapy. Patients without biopsies (*n* = 1), tissue specimens without sufficient tumor epithelial cells (*n* = 5) for nucleic acid extraction, or insufficient DNA for OncoScan (*n* = 10) were excluded from the analyses (Figure 1). We identified 14 IBC patients with medical records data and FFPE breast biopsy specimens eligible for multi-omic assessment.

### 2.2. TCGA Study Population

We identified non-inflammatory breast cancer samples in The Cancer Genome Atlas (TCGA) CNV data [20]. TCGA contains data on breast ductal and lobular tumor samples assayed on several platforms. CNV data were obtained from ‘CopyNumber Gistic2′. The GISTIC 2.0 software was used to identify regions of genes with significant amplification or deletion [21]. The selection of TNBC cases and their classification was adopted from established schema [20]. Our analysis compared the 14 DFCI IBC stage III patients and 388 stage III TCGA non-IBC patients. The analyses were stratified by molecular subtype.

### 2.3. Pathology Review and IBC Specimen Preparation

The FFPE core biopsy specimens underwent centralized pathology review at Brigham and Women’s Hospital. Sections of 4 μM were prepared from paraffin-embedded clinical biopsy specimens and stained for H&E. Pathology review identified tumor, where % tumor cellularity exceeded 30% of the region of interest (ROI). The length of the biopsied material was also estimated to determine how feasible scraping of ROI would be for subsequent downstream DNA and RNA isolation. Lymphovascular invasion and/or dermal lymphatic invasion (DLI) were noted. For up to 15 patients, 4 μM unstained sections were then scraped utilizing the annotated H&Es to match the ROI for each case.

### 2.4. Dual DNA/RNA Extraction from Paraffin-Embedded IBC Biopsy Material

Nucleic acid was extracted from 14 IBC biopsy specimens. All scrapings for a given case were pooled and the Qiagen AllPrep DNA/RNA FFPE Kit (Qiagen-cat# 80234) following the manual’s instructions was utilized for subsequent RNA extraction. We included DNA and RNA extracted from an IBC cell line (SUM149 cells) and technical replicates for quality control assessment. The xylene/ethanol deparaffinization method for upstream processing of the samples was implemented. The scraped paraffin-embedded tissue is dissolved in 99–100% xylene. After precipitation of the sample and removal of the supernatant, residual xylene is removed by washing with 96–100% ethanol. Briefly, once the paraffin has been removed, tissue is lysed with proteinase K digestion. After incubation at 4 °C and centrifugation, the RNA-containing supernatants and DNA-containing pellet are separated and undergo independent processing. RNA supernatant is incubated at 80 °C and then RNA is bound to the membrane of the RNeasy MinElute spin column and treated with DNase, then subjected to a series of wash steps, prior to elution in a 14 μL volume of RNase/DNase-free H_2_O. The DNA-containing pellet is lysed with proteinase K digestion and incubated at 90 °C. DNA is bound to the membrane of the QIAamp MinElute spin column and subjected to a series of wash steps, prior to elution in a 30 μL volume of RNase/DNase-free H_2_O.

### 2.5. Quantification of RNA and DNA from Formalin-Fixed Paraffin-Embedded (FFPE) IBC Biopsy Material

RNA and DNA isolates were quantified utilizing the Quant-iT RiboGreen assay (Life Technologies-cat# R11490) and Quanti-iT PicoGreen assay (Life Technologies-cat# P7589), respectively. A total of 1 μL of RNA and RNA is required for quantification. Concentration is measured as ng/ul. For RNA quantification, isolates were excited at 485 ± 10 nm and the fluorescence emission intensity was measured at 530 ± 12 nm using a Victor X3 spectrophotometer (Perkin Elmer cat# 2030-0030). Fluorescence intensity was plotted versus RNA concentration over the calibration range, 0–100 ng/μL. For DNA quantification, isolates were excited at 480 nm and the fluorescence emission intensity was measured at 520 nm. Fluorescence intensity was plotted versus DNA concentration over the low calibration range, 0–50 ng/μL.

### 2.6. Human Transcriptome Array

Sensation Plus FFPE Amplification protocol was used to amplify 50 ng of RNA from the biopsies and profiled using the Affymetrix Human Transcriptome Array (HTA) 2.0. The HTA technology allows comprehensive examination of gene expression and genome-wide identification of alternative splicing as well as detection of noncoding transcripts in FFPE tissue [22]. There are probes for gene exons, exon–exon junctions, coding SNPs, noncoding RNAs, and noncoding antisense RNAs. The HTA 2.0 includes 44,000 mRNA transcript clusters and 22,000 lncRNA transcripts. We used robust multi-array average (RMA) normalization of transcript levels.

### 2.7. OncoScan Gene Chip

The extracted DNA was analyzed with the Affymetrix OncoScan platform (Santa Clara, CA, USA). This assay utilizes the Molecular Inversion Probe (MIP) assay technology for the detection of single nucleotide polymorphism genotyping, insertions, deletions, large fragment copy number variation (CNV), loss of heterozygosity (LOH), and somatic mutation. The CEL files were obtained by the Affymetrix GeneChip Scanner and converted to the OSCHP files by Chromosome Analysis Suite 3.2 (ChAS) software (ThermoFisher Scientific, Waltham, MA, USA). Log ratio (R) represents the total signal intensity, which reflects the total copy number on a logarithmic scale. The B-allele frequency (BAF) represents the allelic contrast and demonstrates the relative presence at each SNP locus evaluated of the two alternative nucleotides. ChAS and Nexus 10.0 Copy Number Discovery software included in the Affymetrix OncoScan FFPE Express Service (Bionano Genomics, Nexus Copy Number, San Diego, CA, USA) were used to evaluate the IBC genome. The OncoScan GeneChip includes 875 gene targets representing tumor suppressor genes and oncogenes, each gene is represented by 20–40 probes depending on the length of the gene. OncoScan performance requires 80 ng of DNA; however, several of our samples had lower DNA yield.

### 2.8. Quality Control and Biological Concordance Analysis

Pearson correlation coefficients for CNV and overall gene expression were used to evaluate technical replicates (IBC patient biopsies and SUM149 cell line) and demonstrated excellent reproducibility (*r* = 0.96) in biopsy tissue. Biological concordance of estrogen receptor (ER) immunohistochemistry (IHC) levels and estrogen receptor 1-alpha (ESR1-α) mRNA was observed for ER+ patients (Supplementary Figure S1a). In the CNV chromatogram, amplification of the ERBB2 gene was observed in the HER2-type IBC patient (Supplementary Figure S1b). We conducted principal component analysis (Supplementary Figure S2).

### 2.9. Statistical Analysis

**Figure 2:**
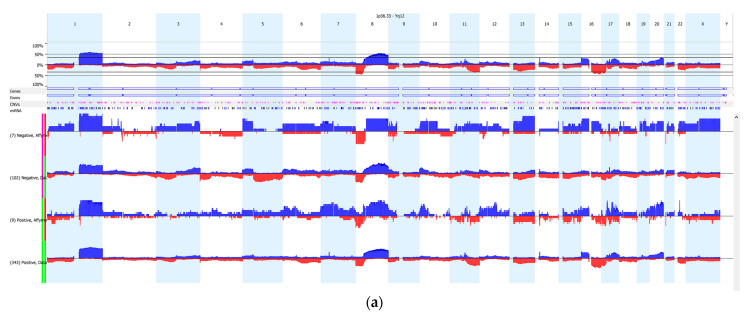

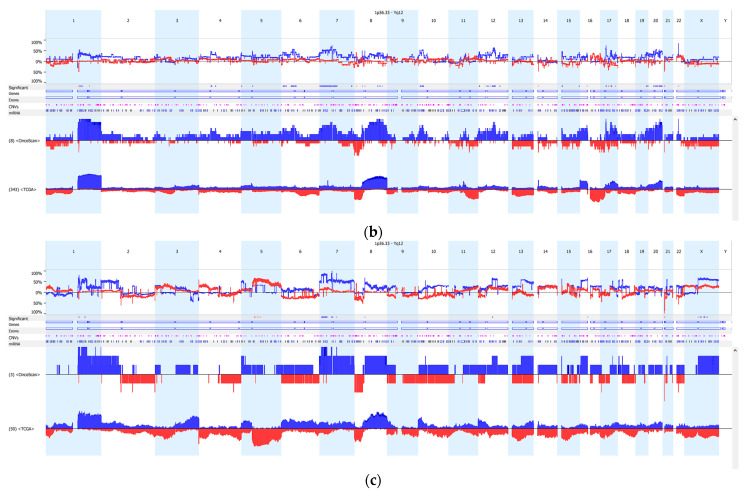
CNV-ome in inflammatory breast cancer compared to non-inflammatory breast cancer patients. Segmental chromosomal aberrations in each chromosome arm (losses of genomic material are in red, and gains in blue) are presented by receptor status. (**a**) Analyses of CNV data in 14 IBC patients compared to CNV data in 388 non-IBC patients showed enrichment for gain on chromosome 7 and loss on chromosome 21 in IBC samples (*p* < 5.0 × 10^−5^). (**b**) Analyses of CNV data in 8 ER+ IBC patients compared to CNV data in 338 non-IBC patients showed enrichment for gain on chromosome 7 and loss on chromosomes 1 and 21 in IBC samples (*p*-value = 3.12 × 10^−10^). (**c**) Analyses of CNV data in 3 TN-IBC patients compared to CNV data in 50 non-IBC patients showed enrichment for gain on chromosome 7 and loss on chromosome 21 in IBC samples (*p*-value = 4.27 × 10^−5^).

The Affymetrix Expression Console and TAC software were used to conduct quality control analyses (described above). In a secondary analysis, we explored the IBC transcriptome in samples prior to systemic treatment. Differential expression analyses were conducted using one-way ANOVA with Tukey’s HSD correction. Log-fold changes were used to evaluate transcriptome profiles by CNV status. The False Discovery Rate (FDR) was used to correct multiple testing. Nexus and ChAS software were used to evaluate copy number data for IBC patients. Figure 2 was generated using Nexus Copy Number.

Chi-square and Fischer’s exact test (categorical variables) and *t*-test (continuous variables) or the Wilcoxon signed-rank test were used to assess demographic characteristics. Descriptive analyses were conducted using R programming (version 3.7). ER expression by IHC analysis was used to stratify patient data. Triple-negative IBC (TN-IBC) was defined as ER-negative (ER-), progesterone-negative (PR-), and HER2-negative (HER2-). Two-sided *p*-values less than 0.05, after multiple testing correction (FDR), were considered statistically significant.

## 3. Results

### 3.1. Inflammatory Breast Cancer Patients

The demographic and clinicopathological data of the 14 women diagnosed with IBC are summarized in Table 1. The mean age at diagnosis was 53.3 years. All tumors were grade 2 (*n* = 4) or grade 3 (*n* = 10). The subtype distribution was the following: ER+/HER2- in *n* = 10, HER2+ in *n* = 1, and ER-/PR-/HER2- in *n* = 3. We observed DLI in 60% of ER+ patients and 25% of ER- patients. The mean body mass index (BMI) was 29.1 (standard deviation, std., ±11.2) in ER+ and 26.5 (std. ±3.4) in ER- patients.

### 3.2. Copy Number Variation (CNV)

Next, we compared CNV changes in 14 IBC patients and 388 non-IBC patients (TCGA) by receptor status. The CNV data were filtered to include changes detected in at least 35% or a greater proportion of the samples. This CNV analysis showed that significant gain on chromosome 7 (p11.2-11.1) was identified in 14 IBC patients (Figure 2, Table 2) compared to 388 non-IBC patients. Stratified by subtype, the CN gain was identified in 6 out of 8 ER+ IBC patients (chr7: 58,019,983–58,025,423) vs. 199 out of 338 ER+ non-IBC patients (FDR *p*-value = 3.12 × 10^−10^) and 3 out of 3 TN-IBC patients (chr7: 57,950,944–58,025,423) vs. 0 out of 50 TN non-IBC patients (FDR *p*-value = 4.27 × 10^−5^). The strongest signal, after multiple testing correction was for CN gain, on 7p11.2-11.1 near the *EGFR* gene. *EGFR* mutations (e.g., *EGFR* exon 19: 55,242,465–55,242,479) were also observed in IBC patients compared to non-IBC patients (FDR *p*-value = 0.00894 for TN-IBC and FDR *p*-value = 0.023 for ER+ IBC). CNV loss was observed on chromosome 21p11 (9,648,315–9,764,385) for 4 out of 8 ER+ IBC patients (FDR *p*-value = 1.13 × 10^−7^) and 3 out of 3 TN-IBC patients (FDR *p*-value = 4.27 × 10^−5^) compared to 0 out 388 non-IBC patients (Figure 2b–c, Table 2). CNV loss on chromosome 1q was observed in 4 out of 8 ER+ IBC patients compared to 99 out of 338 ER+ non-IBC patients (FDR *p*-value = 5.58 × 10^−7^).

**Figure 3:**
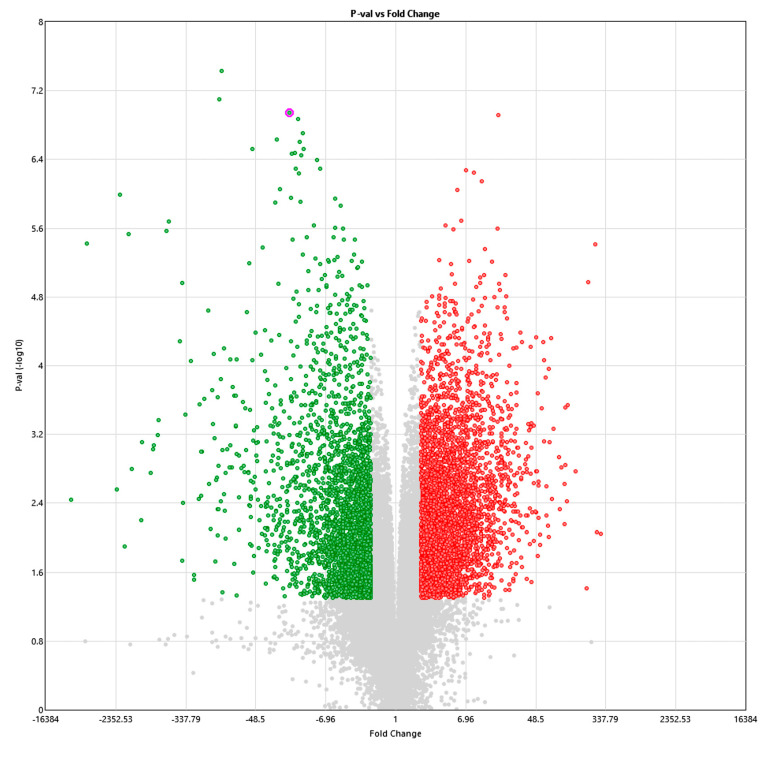
Volcano Plot of *p*-value vs. fold change (red: up 3688 genes; green: down 3263 genes) for TN-IBC samples with chromosome 7 CN gain compared to SUM149 cell line.

**Figure 4:**
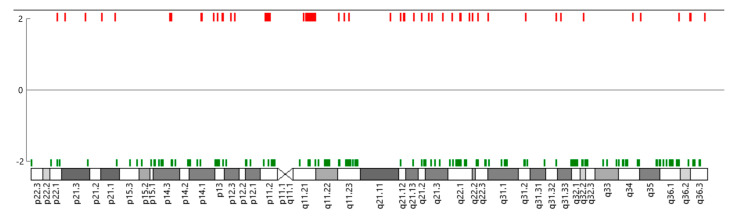
Exploratory differential gene expression (DGE) on chromosome 7 (red = up; green = down; DGE results for genes with an FDR *p*-value < 0.0199) in IBC patients with Chr 7 CN gain compared to SUM149.

Differential gene expression in IBC patients with CN alterations compared to the SUM149 cell line was observed but should be interpreted with caution due to the small sample size and multiple testing (Figure 3 and Figure 4).

Although limited by sample size, we explored differential gene expression on chromosome 7 in IBC patients with 7p11.2 CN gain compared to SUM149 (Figure 4). The strongest signal on chromosome 7 was in an unannotated probe (HTA 2.0 probe ID: TC07001784.hg.1, fold change = −15.92, FDR *p*-value = 0.0016). Differential gene expression was detected (such as TGFB2, CCL2; upregulated genes = 394, downregulated genes = 169) but was not statistically significant after FDR correction and filtering. EGFR coding mRNA levels, on chromosome 7p11.2, in patients with CN gain were not significantly higher compared to SUM149 levels (fold change = 6.63; FDR *p*-value = 0.1096, data not shown).

## 4. Discussion

CNV describes a class of alterations where a small or large region of genetic material is gained or lost. Copy number variation has been detected in IBC [8,15,16,23,24,25,26,27,28] and CN amplification has been reported in profiling studies on chromosomes 1q, 8q, and 17q. Previous copy number studies reported amplification of the anaplastic lymphoma kinase (ALK) gene on chromosome 2 in IBC patients [16,29,30]. In this observational study, we evaluated CNV changes in IBC specimens collected prior to systemic therapy and compared CNV alterations in IBC patients with non-IBC patients. We observed a statistically significant CN loss on chromosome 1q in ER-positive IBC patients compared to non-IBC patients (Table 2). The suggested CN gain on chromosome 8q was observed in TN-IBC (FDR *p*-value = 0.002, data not shown). We observed novel enrichment for CN gain on chromosome 7p11.2-p11.1 for 75% of ER+ IBC patients and all TN-IBC patients compared to non-IBC patients by receptor status (Table 2). The strongest signal, after multiple testing correction, was for CN gain on 7p11.2-11.1 near the *EGFR* gene (Table 2). *EGFR* mutations (e.g., *EGFR* exon 19: 55,242,465–55,242,479) were also observed in IBC patients compared to non-IBC patients (FDR *p*-value = 0.00894 for TN-IBC and FDR *p*-value = 0.023 for ER+ IBC). Higher EGFR protein levels have been reported in breast cancer, especially in TNBC and IBC [31,32,33].

Preclinical [34] and clinical data [35] indicate that EGFR tyrosine kinase inhibitor may be a promising therapeutic target for IBC [36]. A single-arm phase 2 study demonstrated that an anti-EGFR antibody, panitumumab, combined with neoadjuvant chemotherapy, had significant clinical activity in HER2-negative IBC, particularly in TN-IBC patients (NCT01036087; panitumumab combined with neoadjuvant chemotherapy resulted in the highest pathological complete response (pCR) rate in 8 out of 19 TN-IBC patients) [37]. In this trial, pretreatment EGFR tumor marker (protein) expression, but not EGFR mRNA expression, was a prognostic marker in TN-IBC. Three patients with pCR had an increase in CD8+ T cells and a decrease in Tregs and M2 macrophages after treatment with panitumumab. An ongoing clinical trial (NCT05177796) is evaluating the enhancement of immunotherapy by targeting the EGFR pathway with panitumumab and pembrolizumab in combination with neoadjuvant chemotherapy in TN-IBC. In a humanized mouse model study, panitumumab remodeled the IBC tumor microenvironment (TME) by increasing cytotoxic T cells and reducing immunosuppressive regulatory T cells and M2 macrophages [38]. Furthermore, panitumumab reduced the gene expression of immunosuppressive cytokines, including TGFB1, TGFB2, TGFB3, CCL2, and IL1B [38]. EGFR targeted therapy has been shown to modulate the TME in lung cancer [39,40].

We also observed CN loss on chromosome 21p11.2 in ER+ IBC and TN-IBC patients compared to non-IBC patients. Melanoma antigen (BAGE) on chromosome 21p11.1 has been associated with cancer [41]. Loss of 21p11.2-p11.1 has been observed in breast cancer patients with pathogenic ataxia telangiectasia mutated (ATM) gene variants [41]. Copy number gain of chromosome 7p21.1 and loss of chromosome 21p11.1 co-occurred in gastric cancer [42].

We previously validated the utility of breast FFPE tumor samples for expression analysis through comparative differential expression analysis of TCGA fresh-frozen samples [43]. In the current study, our quality control data demonstrated that simultaneous DNA and RNA extraction and multi-omic assessment of FFPE core biopsy tissue from IBC patients are robust and reproducible (*r* = 0.96). A strength of this study is the important selection of samples before systemic treatment.

Our study has several limitations. First, there was a small sample size of eligible IBC tissue samples. Second, the DNA yield for some samples was below the required amount for the OncoScan platform. Third, we did not have EGFR or TME immunohistochemistry data for these IBC patients. Fourth, CNV changes in the DFCI IBC FFPE samples (OncoScan) and TCGA non-IBC fresh-frozen samples were evaluated on different platforms with variable probe coverage. The analyses of copy number changes in IBC significantly associated with mRNA, miRNA, or lncRNA changes were limited by sample size and gaps in the annotation. Fifth, the analyses may be susceptible to unaccounted bias. A larger study is warranted to evaluate paired CNV and TME changes by IBC subtype.

## 5. Conclusions

In conclusion, this small study showed chromosome 7 gain and chromosome 21 loss in biopsy samples collected prior to initiating preoperative systemic treatment in IBC patients compared to non-IBC patients. Although limited by sample size, these data suggest that CNVs may contribute to biological heterogeneity. The hypothesis generated warrants further investigation in a larger dataset.

## Figures and Tables

**Table 1:** Characteristics of Inflammatory Breast Cancer Patients by Estrogen Receptor Status.

	ER + IBC (*N* = 10)	ER − IBC (*N* = 4)
	*N* (%)	*N* (%)
Age at diagnosis, Mean years (SD)	53.3 (6.7)	53.5 (4.0)
Grade		
II	3 (30)	1 (25)
III	7 (70)	3 (75)
% Dermal Lymphatic Invasion	6 (60)	1 (25)
% Family History of Breast Cancer	6 (60)	0 (0)
Body Mass Index, Mean kg/m^2^ (SD)	29.1 (11.2)	26.5 (3.4)

**Table 2:** CNVs in Inflammatory Breast Cancer Compared to Non-Inflammatory Breast Cancer (TCGA data) by Subtype.

Region	Cytoband Location	Event	Region Length **	Frequency in IBC (%) *	Frequency in non-IBC (%)*	Difference	*p*-Value
**ER+ (n=8 IBC, 343 non-IBC)**							
chr7:58,019,983-58,025,423	p11.1	CN Gain	5440	75	0	75	1.13 × 10^−11^
chr7:57,950,944-58,019,983	p11.2–p11.1	CN Gain	69039	75	0.58	74.42	3.12 × 10^−10^
chr21:9,648,315-9,764,385	p11.2	CN Loss	116070	50	0	50	1.13 × 10^−07^
chr1:149,248,784-149,293,460	q21.2	CN Loss	44676	50	0.29	49.71	5.58 × 10^−07^
**TN (n=3 IBC, 50 non-IBC)**							
chr7:57,950,944-58,025,423	p11.2–p11.1	CN Gain	74479	100	0	100	4.27 × 10^−05^
chr21:9,648,315-9,764,385	p11.2	CN Loss	116070	100	0	100	4.27 × 10^−05^

* Data were filtered to show CNVs detected in at least 35% or greater proportion of samples. ** 100% of CNV region overlap for IBC and non-IBC patients.

## Data Availability

The TCGA-BRCA data are available at the NCI Genomic Data Commons (https://portal.gdc.cancer.gov). The TCGA-BRCA dataset accession number is phs000178. Aggregate results have been included in tables while stewarding data privacy and security for rare IBC outcomes. IBC HTA 2.0 gene expression data has been uploaded in NCBI GEO (Accession Number GSE228601).

## Supplementary Materials

The following supporting information can be downloaded at: https://www.mdpi.com/article/10.3390/cells12071086/s1, Figure S1: IBC CNV Chromatogram; Figure S2: Principal component analysis (PCA) of TN-IBC samples compared to SUM149 cell line.
