# Supplementary material for: Copy Number Variation in Inflammatory Breast Cancer
Source: Cells. 2023 Apr 4;12(7):1086. doi: 10.3390/cells12071086 (PMC10093603; doi:10.3390/cells12071086)
Supplement: Supplementary file 1 [file cells-12-01086-s001.zip › cells-2245016-supplementary.pdf]

Supplementary Materials

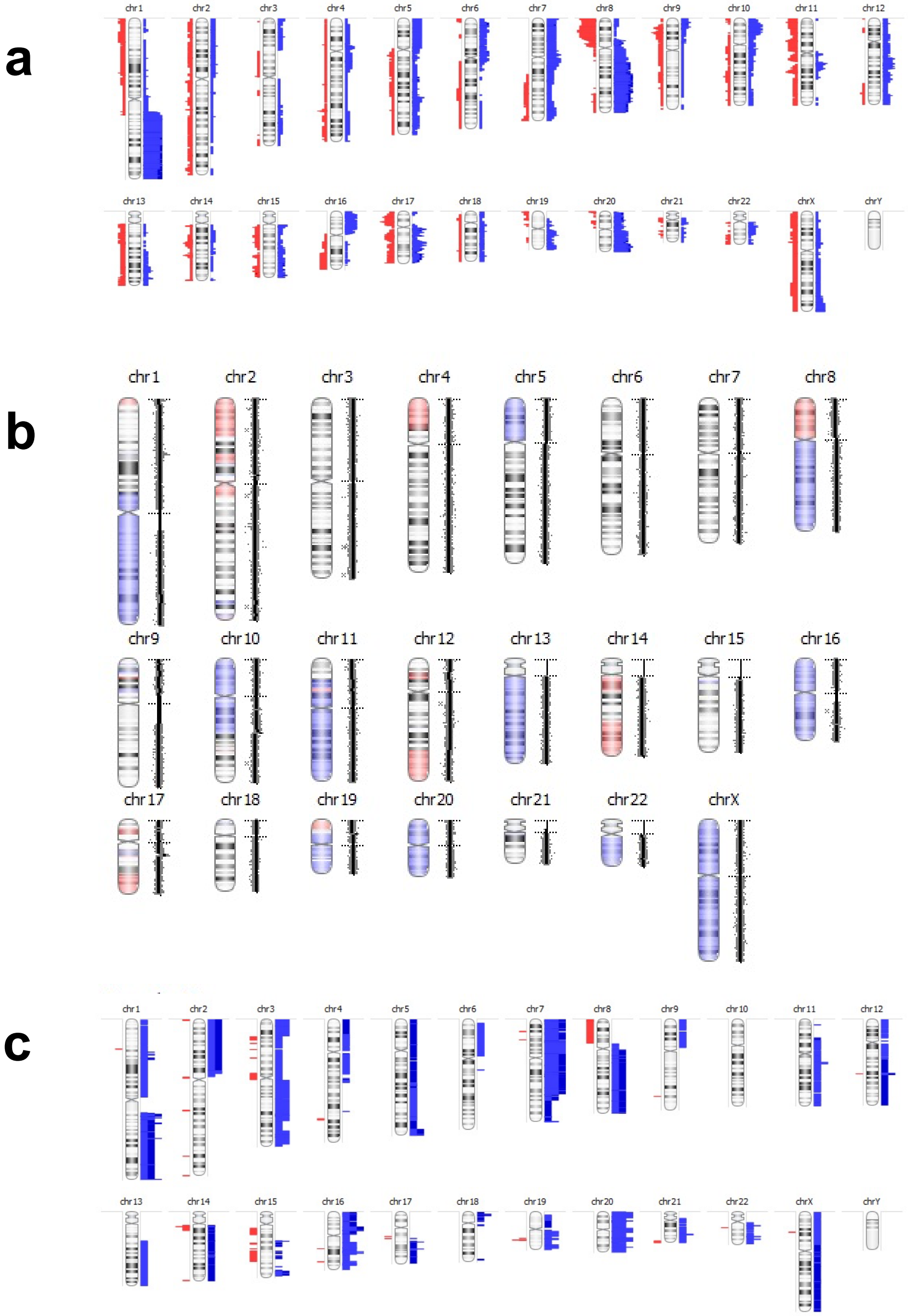

Figure S1. IBC CNV Chromatogram. (**a**) CNV Chromatogram for ER-positive IBC patients (n=8). (**b**) CNV Chromatogram HER2 Type IBC patient (n=1). (**c**) CNV Chromatogram TNBC IBC patient (n=3).

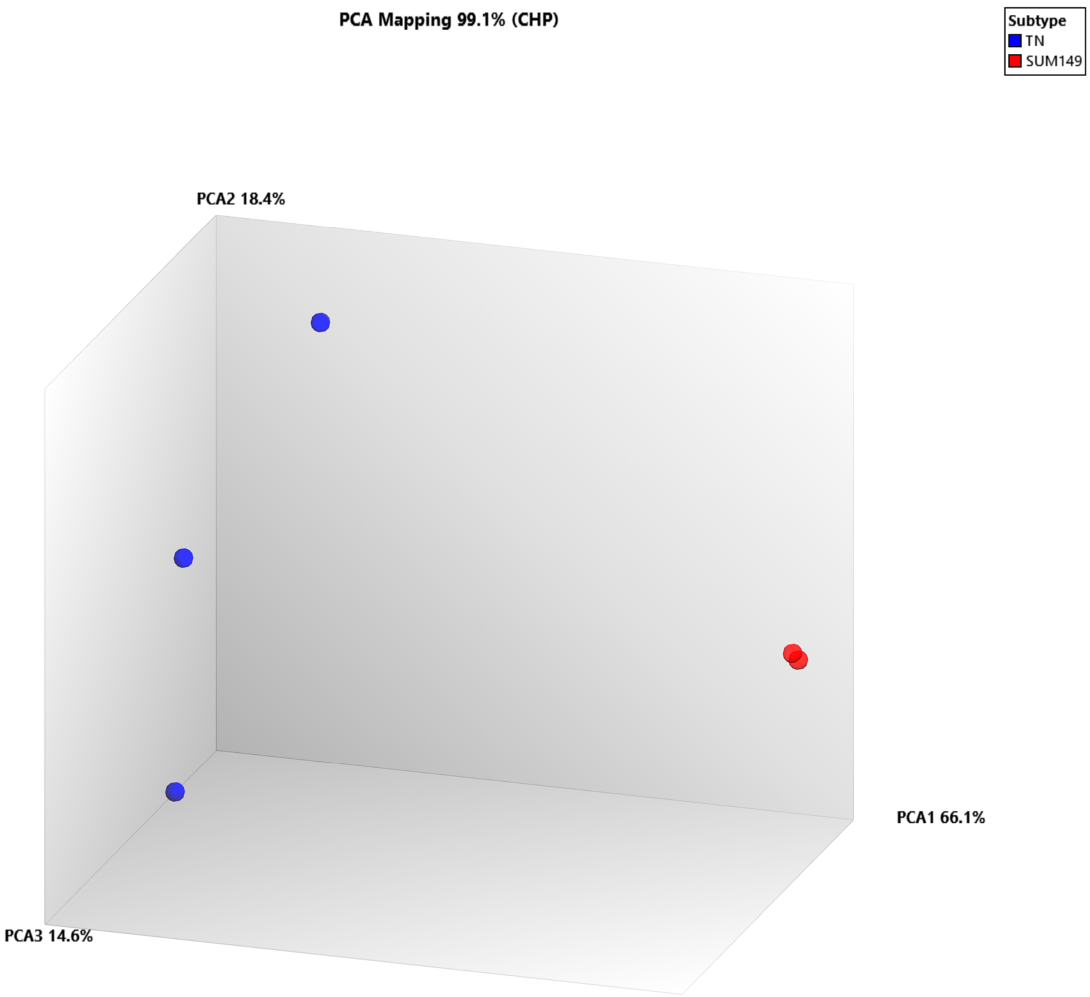

Figure S2. Principal Component Analysis (PCA) of TN-IBC samples compared to SUM149 cell line.
